# MicroRNA‐microbiota interactions: Emerging strategies for modulating intestinal homeostasis and enhancing host health

**DOI:** 10.1002/imo2.57

**Published:** 2025-01-26

**Authors:** Tianle He, Jiaying Ma, Shuai Liu, Boyan Ma, Jingtao You, Jingjun Wang, Mengmeng Li, Wei Wang, Ya Jing Wang, Shengli Li, Zhijun Cao

**Affiliations:** ^1^ State Key Laboratory of Animal Nutrition and Feeding, International Calf and Heifer Organization, College of Animal Science and Technology China Agricultural University Beijing China; ^2^ College of Animal Science and Technology Ningxia University Yinchuan China

**Keywords:** host health, intestinal homeostasis, intestinal immunity, microRNA–microbiota interactions

## Abstract

Long‐term artificial selection and environmental shifts have driven adaptive changes in both the host genome and the intestinal microbiota. The complex symbiotic relationship between these two has become essential for maintaining intestinal homeostasis and overall health. Concurrently, advancements in sequencing technology and the functional annotation of noncoding RNAs, particularly microRNAs (miRNAs), have facilitated the exploration of mechanisms regulating intestinal homeostasis. Herein, we systematically update the role of miRNA–microbiota interactions in regulating the intestinal barrier, intestinal immunity, changes in intestinal microbiota dynamics, and maintenance of intestinal homeostasis, and we further critically discuss the role of miRNA–microbiota interactions in the maintenance of host intestinal health, metabolic regularity, brain function, and neurodegenerative disease‐related disorders. Finally, we highlight the prospects and therapeutic strategies regarding miRNA–microbiota interactions in humans and animals in the context of intestinal microbiota and gene function studies. This study provides a comprehensive overview of miRNA–microbiota interactions and their influence on intestinal homeostasis and host health and offers novel therapeutic strategies for future personalized prevention and treatment of intestinal diseases.

## Highlights

- MicroRNA (miRNA)–microbiota interactions are crucial for maintaining host health by regulating intestinal homeostasis.
- Environmental factors to which the host is exposed can disrupt the delicate balance of miRNA–microbiota interactions and impact overall health.
- Targeting miRNA–microbiota interactions offers a promising avenue for the prevention and treatment of intestinal diseases.
- The search for miRNA–microbiota interaction targets and modes of action are key to manipulating intestinal and host health.

## Graphical abstract

Intestinal homeostasis is crucial for host health. MicroRNA (miRNA)–microbiota interactions maintain intestinal health by regulating the barrier function, immune response, microbiota composition, and their metabolic activities. These interactions further influence host metabolism, behavior, and cognition. With advancements in sequencing and artificial intelligence, precise identification and manipulation of miRNA–microbiota interactions holds promise for manipulating intestinal homeostasis and promoting health in humans and animals.

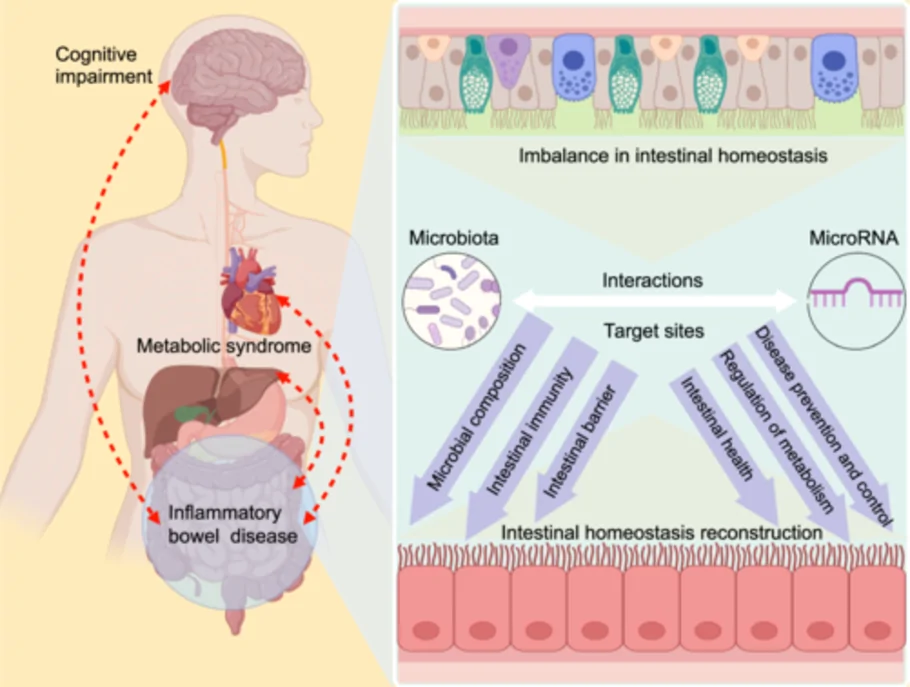

## 1 INTRODUCTION

The intestinal microbiota plays a key role in maintaining the dynamic balance of host physiology by promoting the development of the immune system, regulating host metabolism, and maintaining the health of the host [1]. The distribution and composition of intestinal microbiota depends upon interspecies, intraspecies, and host interactions, resulting in a relatively stable intestinal microbiota environment [2]. In this homeostatic environment, the relative balance between beneficial and harmful bacteria is maintained, and the diversity and function of the flora can function properly. Concurrently, the rapid rise of sequencing technology and functional annotation of noncoding RNAs, particularly microRNAs (miRNAs), have also contributed to the exploration of the regulation of intestinal homeostasis. Previous studies indicated that miRNAs are a class of nucleotide sequences with an average length of 22 bp that primarily regulate physiological processes such as cell proliferation, differentiation, metabolism, and disease progression by modulating the expression of downstream mRNAs [3]. Combining the regulatory role of miRNAs with the formation of intestinal homeostasis will potentially provide novel ideas for analyzing intestinal pathogenesis and identifying therapeutic targets for related diseases.

The importance of intestinal homeostasis has been widely established. Studies from both humans and animals point to intestinal microbiota and miRNA abundance as key to building intestinal homeostasis [4, 5]. The expression level of miRNAs in the host intestine is correlated with the abundance of specific flora [4, 5, 6]. Recent studies demonstrated that miRNAs could affect the diversity of intestinal microbiota by regulating the expression of host and microbiota genes, and this ultimately alters the composition of intestinal microbiota [4, 5, 6]. This regulation of gene expression is an important mechanism whereby the host resists disorders of the intestinal microbiota [5, 6]. Intestinal tissues and microbiota can release miRNAs directly (or indirectly by secreting exosomes) and deliver them to host intestinal tissues that, in turn, participate in the gene expression regulation process and play a vital role in host intestinal homeostasis and physiological, immune, and metabolic functions [7, 8]. Additionally, intestinal microbiota can stimulate the host immune response through the production of metabolites or cellular components to further influence the expression and function of miRNAs [9]. Therefore, the interaction between miRNAs and microbiota may provide an important contribution to the construction and regulation of intestinal homeostasis. Notably, the study of the interactions between different miRNAs‐microbiota should not only consider their own attributes but also consider other factors that may affect the interactions between the two.

Although the importance of the abundance and diversity of the intestinal microbiota and the dynamics of miRNAs in the maintenance of intestinal homeostasis is well established, research investigating intestinal health has focused more on the effects of individual microbiota or miRNAs on intestinal morphology, structure, and function than they have on microbe–miRNA interactions [10]. This is misleading and obstructive to enhancing the treatment of intestinal‐related diseases and understanding the multiple biological roles of the liver‐intestinal, lung‐intestinal, and brain‐intestinal axes. Therefore, an in‐depth understanding of miRNA–microbiota interactions and the importance of these interactions in regulating intestinal homeostasis and host health is essential. This study reviews the formation and mode of action of miRNA–microbiota interactions and further discusses the role and mode of homeostasis formed by miRNA‐microbiota interactions for the regulation of intestinal microbiota, intestinal barrier construction, intestinal inflammation, host metabolism, and cognitive functions of the brain, and based on this, outlines the miRNA–microbiota interactions mechanism in the diagnosis and treatment of intestinal‐related diseases and indicates the potential clinical applications.

## 2 FORMATION OF miRNA–MICROBIOTA INTERACTIONS AND THEIR ROLE IN MAINTAINING INTESTINAL HOMEOSTASIS MECHANISMS

The intestinal microbiota in both humans and animals is initially obtained from the maternal microbiota and stabilizes over time. It can be influenced by factors such as environmental settings, health conditions, and dietary choices [11]. The microbiota plays a pivotal role in modulating the expression and function of host intestinal miRNAs by releasing metabolic products and bacterial exosomes. The sustained interaction between the microbiota and host miRNAs is essential for the maintenance of intestinal equilibrium [12, 13]. Furthermore, the significance of intestinal homeostasis in relation to intestinal morphology, structural integrity, functional capabilities, and overall host health has garnered considerable attention in the current scientific literature [14, 15, 16, 17, 18, 19, 20, 21, 22, 23, 24, 25, 26, 27, 28, 29, 30, 31, 32, 33, 34, 35, 36, 37, 38, 39, 40, 41, 42, 43, 44, 45, 46, 47, 48, 49, 50, 51, 52, 53, 54, 55, 56] (Table 1).

**TABLE 1:** Effects of microRNA (miRNA)–microbiota interactions on intestinal homeostasis.

MiRNAs	Targets	Contributing factors	Action mode	Positive/negative regulation^a^	Reference
miR‐195‐5p	Claudin‐1 (CLDN1), Claudin‐2 (CLDN2)	Tight Junctions (TJs)	Modulation of the intestinal barrier structure	﹢	[15]
miR‐122a	Occludin (OCLN), Zona Occludens 1 (ZO‐1)	TJs	Modulation of the intestinal barrier structure	﹢	[16]
miR‐181a	OCLN, ZO‐1	TJs, Inflammatory factor	Modulation of intestinal barrier structure, immune and inflammatory responses	﹢	[17]
miR‐320a	Junctional adhesion molecule A (JAM‐A)	TJs	Modulation of the intestinal barrier structure	﹢	[18]
miR‐7a	ZO‐1, OCLN, Claudin‐4 (CLDN4), Mucin 2 (Muc2)	TJs, Muc2 and Estimated Glomerular Filtration Rate (EGFR)	Modulation of the intestinal barrier structure	﹢	[19]
miR‐150	ZO‐1, OCLN, CLDN4, Muc2	TJs, Muc2, and intestinal epithelial cell (IEC)	Modulation of the intestinal barrier structure	﹢	[20, 21]
miR‐219a‐5p	Epithelial cadherin (E‐Cadherin)	TJs	Modulation of intestinal barrier structure and inflammatory response	﹢	[22]
miR‐200c‐3p	—	IEC	Modulation of the intestinal barrier structure	﹢	[23]
miR‐374a‐5p	—	Inflammatory factor, IEC	Modulation of intestinal barrier structure and inflammatory response	﹢	[24]
miR‐181c	—	Inflammatory factor and IEC	Modulation of intestinal barrier structure and inflammatory response	﹢	[25]
miR‐381‐3p	—	IEC	Modulation of the intestinal barrier structure	﹢	[26]
miR‐324‐5p	—	IEC	Modulation of the intestinal barrier structure	﹢	[27]
miR‐146b	—	Inflammatory factor	Modulation of intestinal immune and inflammatory responses	﹢	[28]
miR‐766‐3p	Free Alongside Ship (Fas)	Inflammatory factor	Modulation of intestinal barrier structure and inflammatory response	+	[29]
miR‐135a‐5p	Just Another Kinase 2 (JAK2)	Enzyme activity	Modulation of the intestinal barrier structure	+	[30]
miR‐29	Nucleotide‐binding oligomerization domain 2 (NOD2)	Inflammatory factor	Modulation of intestinal barrier structure and inflammatory response	−	[31, 32]
miR‐29a	ZO‐1, CLDN1	TJs	Modulation of the intestinal barrier structure	−	[33]
miR‐29b	ZO‐1, CLDN1	TJs	Modulation of the intestinal barrier structure	−	[34, 35]
miR‐423‐5p	TJs	Inflammatory factor	Modulation of intestinal immune and inflammatory responses	−	[36]
miR‐31	—	T cells and oxidative stress	Regulation of the intestinal barrier, intestinal immunity, and inflammatory response	−	[37, 38]
miR‐146a	—	IEC	Modulation of intestinal immune and inflammatory responses	−	[39]
miR‐21	Metadherin (MTDH)	T cells, Inflammatory factors, and transcriptional activity	Modulation of intestinal barrier structure and inflammatory response	−	[40, 41]
miR‐155	Suppressor of Cytokine Signaling 1 (SOCS1)	NLR family pyrin domain containing 3 (NLRP3)	Modulation of intestinal barrier structure and inflammatory response	−	[42]
miR‐212	—	Inflammatory factor	Modulation of intestinal barrier structure and inflammatory response	−	[43]
miR‐133a‐3p	—	IEC	Modulation of the intestinal barrier structure	−	[27]
miR‐125b‐5p	—	IEC	Modulation of the intestinal barrier structure	−	[44]
miR‐124‐3p	Muc2	Muc2	Modulation of the intestinal barrier structure	−	[45]
miR‐1‐3p	Muc2	Muc2	Modulation of the intestinal barrier structure	−	[45]
miR‐125b	CLDN2	TJs	Modulation of the intestinal barrier structure	−	[46]
miR‐1	CLDN2, ZO‐1	TJs	Modulation of the intestinal barrier structure	−	[45]
miR‐16	CLDN2	TJs	Modulation of the intestinal barrier structure	−	[46]
miR‐144	OCLN, ZO‐1	TJs	Modulation of the intestinal barrier structure	−	[47]
miR‐339‐5p	Vascular Endothelial Growth Factor (VEGFA)	IEC	Regulation of intestinal oxidative stress, cell proliferation	−	[48]
miR‐22	Hexokinase 2 (HK2)	*Interleukin‐6* (*IL‐6*)*/nuclear factor kappa‐B* (*NF‐κB*)	Modulation of intestinal immune and inflammatory responses	−	[49]
miR‐20b‐5p	—	Inflammatory factor	Modulation of the intestinal barrier structure	−	[50]
miR‐93‐3p	Frizzled‐7 (FZD7)	Wnt/β‐Catenin	Modulation of the intestinal barrier structure	−	[51]
miR‐683	Polypyrimidine Tract Binding Protein 1 (PTBP1)	Cell proliferation	Modulation of the intestinal barrier structure	−	[52]
miR‐ 92a‐1‐5p	Hypoxia‐inducible factor alpha (HIF‐1α)	Transcriptional activity	Modulation of the intestinal barrier structure	−	[53]
miR‐195	Doublecortin like kinase 1 (Dclk1)	Transcriptional activity	Modulation of the intestinal barrier structure	−	[54]
miR‐429	Claudin‐8 (CLDN8), ZO‐1, Occludin	Transcriptional activity	Modulation of the intestinal barrier structure	−	[55]
miR‐1236‐3p	Myeloid differentiation primary response protein 88 (MYD88)	Inflammatory factor	Modulation of intestinal immune and inflammatory responses	−	[56]

^a^ “+” indicates positive facilitative regulation and “−” indicates negative inhibitory regulation.

### Origin, dynamics, and function of miRNAs in the intestine

**FIGURE 1:**
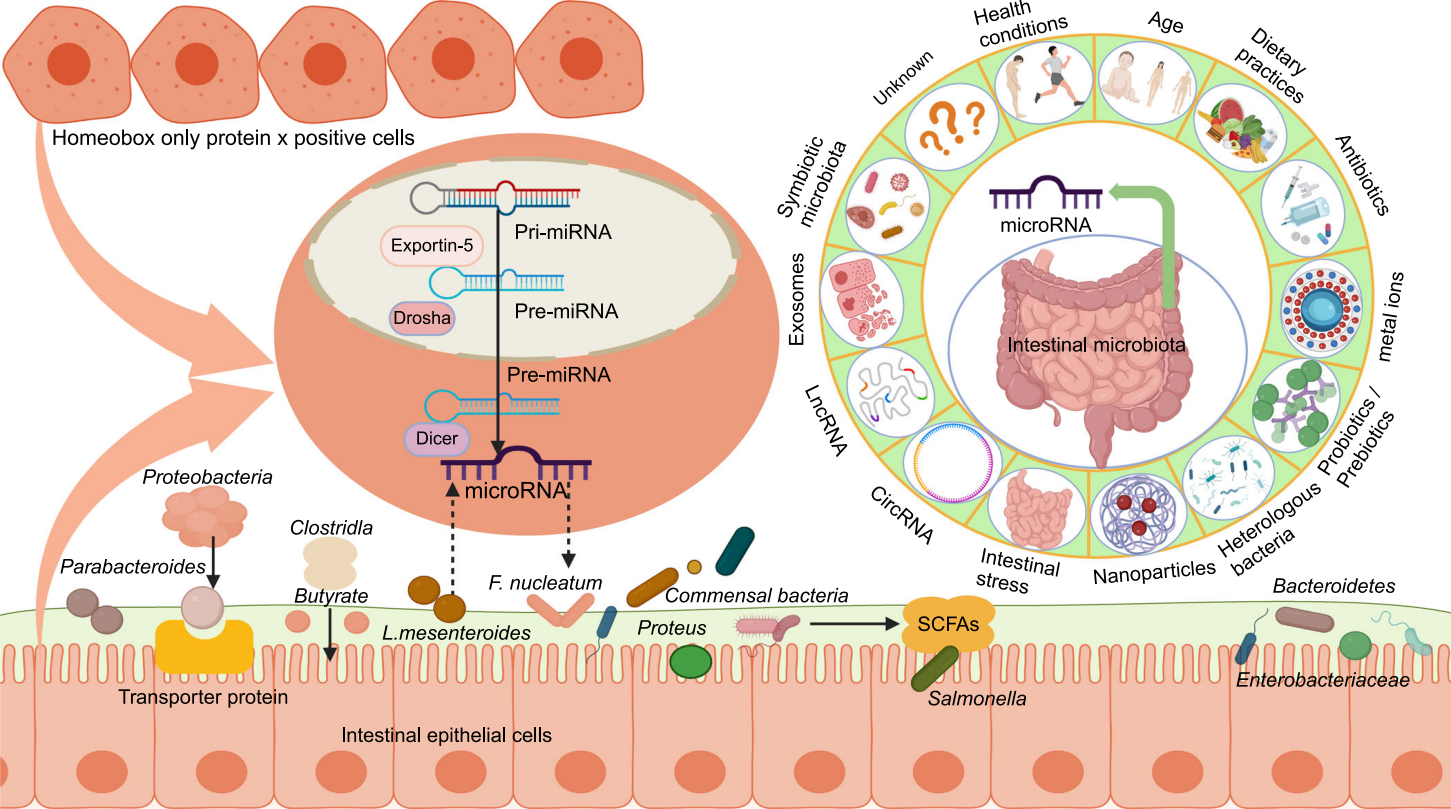
Patterns of microRNA (miRNA) formation in the intestinal tract and factors related to its dynamics. Pathways involved in intestinal miRNA formation include (1) formation by intestinal epithelial cell (IEC) through a transcriptional process and (2) transcriptional formation by positively expressing HOP Homeobox (HOPX) cells. The abundance and variety of miRNAs formed in the intestines in different ways are influenced by a combination of factors, and these miRNAs form complex interactions with intestinal microbiota during long‐term adaptation. Created with Biorender.com. (The solid arrow in the figure represents the direct guiding effect between biomolecules, while the dashed arrow represents the indirect guiding effect between biomolecules).

MiRNAs are noncoding single‐stranded RNA molecules that typically arise from enzymatic cleavage and possess an average length of approximately 22 nucleotides [57]. Within the intestinal, miRNAs are primarily produced by intestinal epithelial cell (IEC) and HOP Homeobox (HOPX)‐positive cells (Figure 1) [5]. Importantly, the types and abundance of miRNAs within this environment are not fixed; rather, they exhibit considerable variability [58]. The dynamics of miRNAs in the intestinal tract are closely connected to several factors, including the age of the host [59], health conditions [10, 60], dietary practices [59], usage of antibiotics [5, 61], and the presence of metal ions [62]. Moreover, the impact of probiotics and prebiotics [63, 64, 65], modifications induced by heterologous bacteria [64], interactions with nanoparticles [66], occurrences of intestinal stress [67, 68, 69], and contributions from circular RNAs [70], long noncoding RNAs [71], exosomes [72], and symbiotic microbiota [73] (Figure 1) all significantly influence the miRNA landscape within the intestine.

### Mode of action of miRNA–microbiota interactions on intestinal homeostasis

The dynamics of intestinal microbiota change in parallel with changes in miRNA species and abundance. Artificial knockout of Dicer1 in mouse IEC and HOPX‐positive cells induces a phenotype of uncontrolled growth of intestinal microbiota. In mice, intestinal microbiota homeostasis can be restored by transplantation of fecal miRNAs from normal mice into knockout mice [5], suggesting that miRNA implantation is involved in the reestablishment of intestinal homeostasis. Donor mouse feces treated with high temperature may also be able to regulate the recipient mouse intestinal microbiota to restore homeostasis [8]. This suggests that miRNAs of host intestinal origin are not only stable but also possess the ability to regulate the homeostasis of intestinal microbiota. Furthermore, studies have conducted an exchange of intestinal microbiota between mice and zebrafish. The results demonstrate that after a certain period of development, the composition of zebrafish intestinal microbiota implanted into mice resembles that of normal zebrafish microbiota. A similar phenomenon was observed in germ‐free mice implanted with zebrafish intestinal microbiota, indicating a regulatory response by host intestinal miRNA within the intestine to maintain intestinal homeostasis and host health [6]. It has been demonstrated that the regulation of intestinal homeostasis by noncoding RNAs is achieved by them entering the cytoplasm of intestinal microbiota after fusion with biofilms, and they are required to target‐bind microbiota messenger RNAs (mRNAs) to regulate microbiota gene expression processes and intestinal homeostasis [60]. Studies using the *Drosophila* animal model have confirmed that certain miRNAs may regulate intestinal bone morphogenetic protein signaling ligands through their target *Cabut* (*Cbt*), and this ultimately results in the regulation of stem cell numbers and intestinal function [69]. Experimental evidence indicates that the establishment or reconstitution of intestinal equilibrium is a consequence of the interplay between the intestinal microbiota and host miRNAs. Regarding the modulatory roles of miRNAs, we delineate three primary mechanisms by which miRNAs govern intestinal homeostasis. First, miRNAs can directly interact with bacterial mRNA through complementary base pairing, resulting in mRNA degradation or the inhibition of its translation, thus impacting the synthesis and functionality of bacterial proteins. Second, miRNAs can modulate bacterial growth and function indirectly by influencing gene expression within host cells. Third, bacteria are capable of secreting or transferring miRNAs to other bacteria, potentially affecting the gene expression and functionality of the recipient bacteria. Furthermore, while miRNAs in bacteria and eukaryotes both modulate gene expression through mRNA complementary pairing, there are notable disparities in their biogenesis and functional mechanisms [60, 61]. This divergence is linked to the observation that eukaryotic miRNAs are typically processed by the enzymes Drosha and Dicer [57], whereas bacterial small RNAs (sRNAs) may be generated and processed through distinct pathways. Additionally, it has come to our attention that the regulatory network of bacterial sRNAs could be more intricate, as they possess the capacity to regulate multiple targets concurrently and are subject to a variety of environmental influences.

## 3 REGULATION OF INTESTINAL HOMEOSTASIS AND INTESTINAL HEALTH BY miRNA–MICROBIOTA INTERACTIONS

### MiRNA–microbiota interactions regulate intestinal barrier function

The intestinal barrier is essential for safeguarding against the invasion of bacteria and toxins, thereby supporting intestinal health and the normal functioning of the immune system [74]. Chronic irregularities in dietary habits, lifestyle choices, and underlying health conditions can significantly compromise the structural integrity of the intestinal barrier, resulting in dysbiosis of the host intestinal microbiota [12, 75]. Existing literature suggests that the interplay between miRNAs and the intestinal microbiota may play a critical role in preserving the integrity and function of the intestinal barrier, thus contributing to the overall health of the host [12]. By comparing mouse intestinal miRNA profiles, Viennois et al. [76] observed that 12 differentially expressed miRNAs interacted with microbiota in the intestines of germ‐free and conventional mice and that sweeteners consumed by colitis mice improved the abundance of intestinal *Mucispirillum* and *Alistipes*, reduced intestinal inflammation, and upregulated the expression of epithelial cadherin (E‐Cadherin) via the miR‐15b/*reversion inducing cysteine‐rich protein with kazal motifs* (*RECK*)/*matrix metalloproteinase‐9* (*MMP‐9*) axis to improve the integrity of the intestinal barrier, ultimately alleviating colitis symptoms [77]. MiRNA–microbiota interactions are the driving factor for maintaining the integrity of the intestinal barrier. In vitro coculture of miRNAs with intestinal microbiota causes hsa‐miR‐515‐5p and has‐miR‐1226‐5p to enter and enhance the proliferative activity of *Fusobacterium nucleatum* and *Escherichia coli*, respectively, and this, in turn, regulates their gene expression and results in selective molecular mechanisms that ultimately manipulate intestinal microbiota composition. Maintaining the intestinal barrier function during intestinal homeostasis depends on its complete physiological structure and complex protein expression, whereas the protein expression in the intra‐and extracellular space of the intestinal barrier layer is also regulated by noncoding RNAs [78, 79, 80], particularly the numerous miRNAs that can target claudins, occluding (OCLN), Zona Occludens 1 (Zo‐1), junctional adhesion molecule, E‐Cadherin, and mucin, thereby regulating the integrity of the intestinal epithelial barrier [15, 18, 23, 45, 46, 68, 81, 82, 83, 84, 85, 86, 87, 88]. MiR‐200b in the mouse intestine reduces the expression levels of myosin light chain kinase (MLCK) and phosphorylated myosin light chain through targeted binding to terminate the redistribution of tight junction proteins in Caco‐2 cells [89]. Similarly, miR‐1 and miR‐185‐3p in the intestines of patients with intestinal inflammation inhibit MLCK expression, ultimately reversing the impaired intestinal barrier function and restoring intestinal homeostasis [82, 90].

The recently proposed theory of intestinal oxidative metabolism and oxidative barrier shaping the composition of the intestinal microbiota explains why exposure of an organism to dietary factors, external factors, and disease can cause oxidative damage to the intestinal barrier that induces a wide range of syndromes in both humans and animals [8] and how the homeostatic environment in which the intestinal microbiota reside and the expression of miRNAs change accordingly when the intestine is in a state of oxidative stress [91, 92]. Further, oxidative stress‐induced damage to the intestinal barrier leads to leakage of oxygen from the intestinal epithelium, and this, in turn, triggers the release of hemoglobin‐carrying oxygen in the mucus layer, thus stimulating the growth of intestinal pathogenic anaerobic bacteria and the rate of anaerobic glycolysis and ultimately leading to an imbalance of intestinal homeostasis and a decline or loss of intestinal microbiota function [93]. Environmental contaminants such as microplastics in certain foods can also cause intestinal microbiota disorders, oxidative damage to the intestinal barrier layer, inflammatory responses, and aberrant miRNA expression [94]. Collectively, these lines of evidence suggest that miRNA–microbiota interactions can regulate the barrier function of the intestine and reveal the importance of miRNA–microbiota interactions in the maintenance of intestinal homeostasis. This information is summarized in Table 1. These findings demonstrate the significance of miRNA–microbiota interactions in the development of intestinal barriers and homeostasis. Additionally, factors such as mucosal molecules from the intestines, lactate, and VFAs from non‐intestinal sources, neurohormones, and sex hormones are also important for facilitating miRNA–microbiota interactions, providing crucial insights for further exploration of intestinal homeostasis.

### MiRNA–microbiota interactions regulate intestinal immunity

**FIGURE 2:**
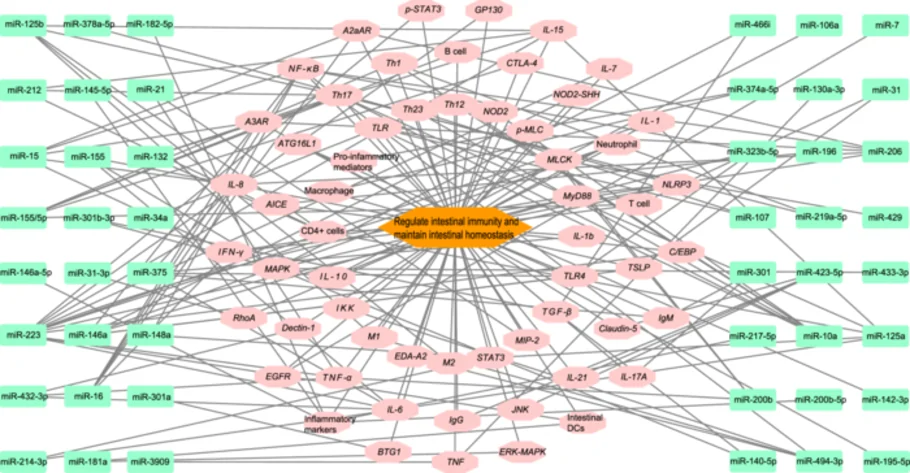
MiRNA–microbiota interactions regulate intestinal immunity. MiRNAs in the intestinal activate intestinal immune responses by targeting inflammatory factors, inflammatory cells, etc., which in turn regulate intestinal homeostasis and host health [19, 38, 98, 99, 100, 102, 104, 105, 106, 107, 108, 109, 110, 111, 112, 113, 114, 115, 116]. Created with Cytoscape 3.10.0.

An intact immune system is essential for the maintenance of intestinal homeostasis [95]. Under normal physiological conditions, intestinal microbiota can contribute to the development of the intestinal immune system and can modulate intestinal development and immune responses through their specific components and intestinal metabolites [96]. For mammals, the interaction between the intestinal microbiota and the intestinal immune system begins at birth, with the intestinal microbiota influencing the development and maturation of the intestinal mucosal immune system and the mucosal immune system being able to shape the composition of the intestinal microbiota [97]. MiRNAs are extremely abundantly expressed in the intestinal mucus layer and IEC and modulate the reception of signals from commensal microbiota or invasive pathogens by IEC that subsequently adapt to altered intestinal environments by modulating the mucosal barrier and signaling to immune cells in the lamina propria [38, 41, 89, 98, 99, 100, 101, 102, 103]. This suggests that miRNAs are not only important for regulating the integrity of the intestinal barrier but are also key inflammatory factors in the intestine and markers of the intestinal inflammatory cascade response. In inflammatory bowel disease (IBD), including Crohn's disease (CD) and ulcerative colitis (UC), the dynamics of a number of these miRNAs (miR‐15, miR‐16, miR‐21, miR‐31, miR‐31‐3p, miR‐125b, miR‐206, miR‐223, miR‐200b, miR‐214‐3p, miR‐146a, miR‐155, and miR‐325) can directly affect immune cell maturation, differentiation, and infiltration, the intestinal immune system, and intestinal homeostasis in conjunction with target chemokines [38, 98, 99, 100, 102, 104, 105, 106, 107, 108, 109, 110] (Figure 2 and Table 1). Certain miRNAs such as miR‐10a, miR‐21, miR‐29, miR‐34a, miR‐107, and miR‐135b act as intermediates between the microbiota and the host immune system of the organism and activate the intestinal immune response, and this, in turn, alleviate the onset of intestinal inflammation and maintains intestinal homeostasis [111, 112, 113, 114, 115, 116] (Figure 2 and Table 1). An important characteristic of miR‐223 that distinguishes it from other miRNAs is that it is able to control the expression profile of interleukin‐1 beta by inhibiting NLR family pyrin domain containing 3 (NLRP3) in the context of UC, leading to an anti‐inflammatory effect [117]. MiRNA–microbiota interactions regulating intestinal immunity are also manifested in terms of immune‐related signaling molecules. Intestinal microbiota can target the Toll‐like receptor (TLR) signaling pathway through negative regulation of miR‐10a that ultimately activates intestinal immune regulation and attenuates inflammatory responses and pathological damage in the intestine [115]. Chassin et al. [118] demonstrated that the upregulation of intestinal miR‐146a expression following colonization of the intestine by a large number of bacteria prevented damage to the intestinal mucosa by bacteria or bacterial lipopolysaccharide, as miR‐146a exerted its protective function through translational repression and protein degradation by acting on TLR‐IL‐1 receptor‐associated kinase (IRAK1). Other studies have suggested that miR‐31 acts as a mediator between the microbiota and intestinal inflammation and that miR‐31 expression induced by changes in microbiota abundance directly activates extracellular pro‐inflammatory signaling formation and inhibits Interleukin‐17 Receptor A (IL17RA) expression in IEC [38]. Notably, miR‐7 can act directly on epidermal growth factor receptors to regulate intestinal immunity by altering the *nuclear factor kappa‐B* (*NF‐κB*), *extracellular regulated protein kinases* (*ERK*), and *protein kinase B* (*AKT*) signaling pathways [19] (Figure 2 and Table 1).

**FIGURE 3:**
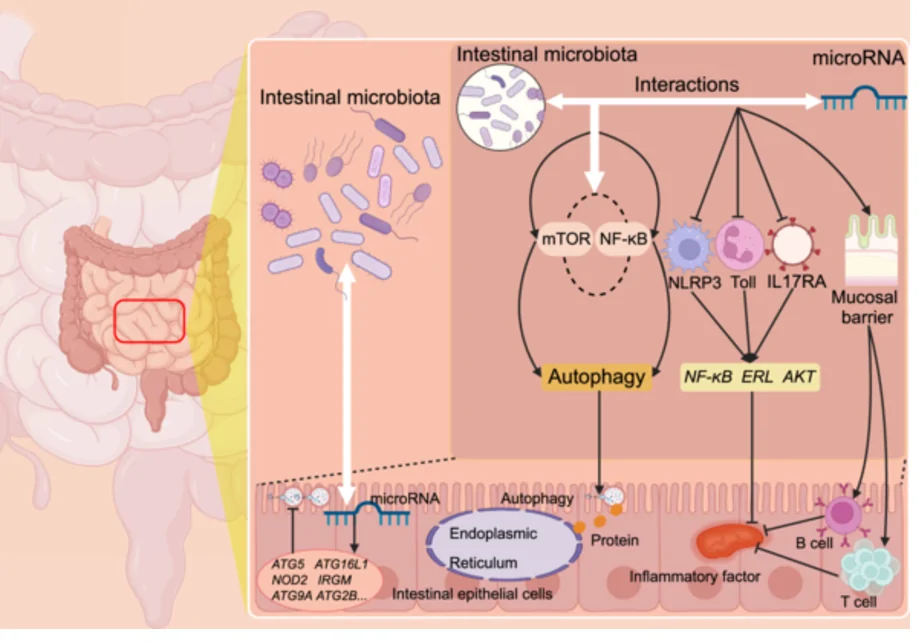
MiRNA–microbiota interactions regulate autophagy in intestinal epithelial cells. MiRNA–microbiota interactions can regulate the autophagy process in IEC in multiple ways, including (1) miRNA target binding to autophagy genes inhibits the intestinal IEC autophagy process but exacerbates intestinal inflammation while disrupting intestinal homeostasis, (2) miRNA–microbiota interactions reduce the level of endoplasmic reticulum protein synthesis and further inhibit intestinal IEC autophagy and disrupt intestinal homeostasis, and (3) miRNA–microbiota interactions regulate IEC autophagy and intestinal homeostasis through the action of intestinal immune pathways (*mammalian target of rapamycin* (*mTOR*), *nuclear factor kappa‐B* (*NF‐κB*), *NLR family pyrin domain containing 3* (*NLRP3*), *Toll*, *Interleukin‐17 Receptor A* (*IL17RA*), *extracellular regulated protein kinases* (*ERK*), and *protein kinase B* (*AKT*)). Created with Biorender.com. (The solid arrow in the figure represents the direct guiding effect between biomolecules, while the dashed arrow represents the indirect guiding effect between biomolecules.)

Additionally, miRNA–microbiota interactions can be involved in the autophagy process of IEC in various manners [4, 12, 114, 119], including target binding of miRNAs to autophagy genes, regulation of endoplasmic reticulum protein synthesis, and modulation of intestinal immune pathways [120]. Autophagy of IEC is an important mechanism for removing harmful intestinal substances, regulating the intestinal immune response, and maintaining the intestinal barrier and intestinal homeostasis [121]. Intestinal infection with adherent‐invasive *E. coli* (*AIEC*) causes upregulation of miR‐30c and miR‐130a that binds to autophagy related 5 (*ATG5)* and autophagy related 16 Like 1 (*ATG16L1)* of IEC through targeting, further inhibiting autophagy and aggravating the intestinal inflammatory response and disrupting intestinal homeostasis [122]. Similarly, miR‐20a, miR‐93, miR‐106a, miR‐106b, miR‐122, miR‐192, miR‐196, miR‐320, and miR‐346 also regulate the expression of *ATG16L1*, *Nucleotide‐binding oligomerization domain 2* (*NOD2*), and *IRGM* and participate in autophagy gene expression [4, 120, 123, 124, 125, 126, 127, 128, 129, 130, 131, 132]. In contrast to miR‐142‐3p, targeting of *ATG16L1* promotes autophagy in IEC and reduces intestinal inflammation [133]. Further, miR‐29a and miR‐143 upregulation inhibit the autophagy process by targeting and regulating the expression of *autophagy related 9A* (*ATG9A*) and *autophagy related 2B* (*ATG2B*) [134, 135]. Additionally, the autophagy process of IEC is associated with the maintenance of endoplasmic reticulum homeostasis and a variety of proteins regulated by miRNAs [136, 137], and the upregulation of certain miRNAs (miR‐150, miR‐375, and miR‐665) induced by intestinal infection with AIEC can lead to a reduction in the levels of proteins synthesized by the endoplasmic reticulum. This ultimately inhibits the autophagy process of IEC induced by intestinal infection [122, 138, 139, 140]. Finally, inflammatory factors involved in intestinal immunity are involved in the autophagy of IEC and maintenance of intestinal homeostasis by regulating *NF‐κB* or *mammalian target of rapamycin* (*mTOR*) signaling pathways in response to miRNAs [141, 142, 143, 144, 145, 146] (Figure 3).

In summary, miRNA–microbiota interactions play a critical role in regulating intestinal immune response, the renewal and replacement of IEC, and maintaining the intestinal barrier and intestinal homeostasis. However, the regulatory mechanisms of miRNAs interacting with the microbiota are not fully understood, providing an opportunity for further exploration. The complexity of the intestinal microbiota complicates the investigation of miRNA–microbiota interactions. Various microbiota species may exert diverse effects on miRNA regulation, potentially antagonizing or synergizing with each other. These intricate interactions make it inappropriate to perceive the role of miRNAs as monolithic. Instead, a more comprehensive research approach and deeper understanding are required. Furthermore, it is important to note the limited research investigating miRNA–microbiota interactions in intestinal immunomodulation and the absence of direct evidence regarding intestinal immune factors as miRNA targets, particularly in animal experiments and clinical studies. This necessitates additional experimental evidence to verify the interactions between miRNAs and the microbiota and their specific effects on intestinal immunity. Addressing these aspects will enhance our understanding and utilization of miRNA–microbiota interactions in the context of intestinal immunomodulation.

### MiRNA–microbiota interactions regulate the structure and diversity of intestinal microbiota

Mouse and human feces contain a large number of miRNAs that are predominantly present in the outer vesicles of IEC [147, 148]. Upon fusion of exosomes with microbiota membrane structures, miR‐515‐5p and miR‐1226‐5p specifically promote the growth of *F. nucleatum* and *E. coli*, thereby disrupting the original colony structure and intestinal homeostasis. Notably, the ability of different miRNAs to enter bacteria differs according to the bacteria [149, 150]. In mice, the abundance of *Parabacteroides* and *Bacteroides* is significantly correlated with intestinal barrier function, whereas the levels of fecal miRNA‐let‐7b and miRNA‐let‐7c of murine origin are negatively correlated with the relative abundance of *Parabacteroides*, and miR‐192 and miR‐194 are negatively correlated with the relative abundance of *Bacteroides* [151]. This suggests that there is an important association between the species and abundance of mouse intestinal microbiota and miRNAs and that this association may be why they are key to maintaining intestinal homeostasis. He et al. [152] reported a significant difference in the composition of miRNAs between the onset and recovery from intestinal inflammation in mice, as evidenced by the significant increase in the level of miRNA‐143a‐3p with the recovery of intestinal inflammation and the growth of *Lactobacillus rohitaensis* through the regulation of its gene expression. MiRNA–microbiota interactions exhibit target specificity for regulating intestinal microbiota structure and diversity, and these interactions are important for regulating intestinal microbiota structure and abundance. Similarly, in IEC‐miRNA‐deficient mice, intestinal microflora was disrupted, thus exacerbating colonic inflammation; however, transplantation of fecal miRNAs from wild‐type mice restored intestinal microflora homeostasis and ameliorated colitis, suggesting that a miRNA deficiency leads to disruption of intestinal microbiota and alteration of the intestinal barrier integrity and that miRNA–microbiota interactions can regulate the balance of intestinal microflora structure, intestinal microbiota diversity, intestinal homeostasis, and host health [153].

## 4 EFFECTS OF miRNA–MICROBIOTA INTERACTIONS ON HOST HEALTH

Recent studies support the important role of intestinal miRNA–microbiota interactions in ecological dysregulation associated with various disease states [154]. The relationship between intestinal oxidation and microbiota is primarily reflected in the metabolism of nutrients by intestinal microbiota, the diversity and abundance of these microbiota, and their impact on intestinal health [155]. Research indicates that patients with IBD experience increased nitrative and oxidative stress in the intestine, resulting in a decrease in the abundance of strict anaerobes from *Bacteroidetes* and *Firmicutes* as well as a reduction in aerobic *Actinobacteria*. Conversely, the abundance of facultative anaerobes from the *Fusobacteria* phylum increases, leading to reduced microbiota diversity, intestinal mucosal damage, and the onset of intestinal inflammation. Furthermore, microbiota‐mediated oxidative stress is significantly associated with gastrointestinal diseases such as colorectal cancer, UC, and CD [156].

**FIGURE 4:**
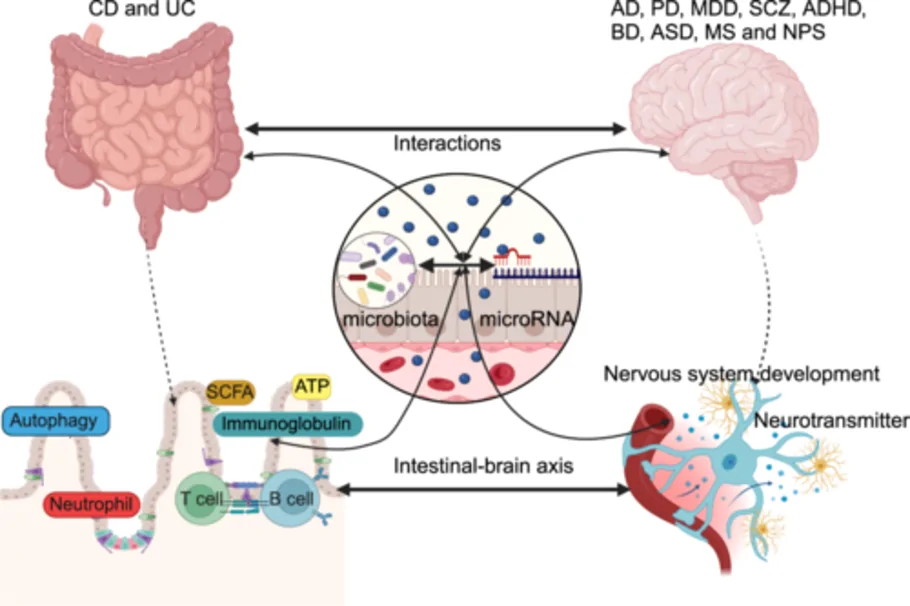
Intestinal miRNA–microbiota interactions in the intestinal‐brain axis. MiRNA‐microbiota interactions in the intestine regulate intestinal diseases and neurological disorders (alzheimer's disease (AD), parkinson disease (PD), major depressive disorder (MDD), schizophrenia (SCZ), attention deficit hyperactivity disorder (ADHD), bipolardisorder (BD), autism spectrum disorder (ASD), multiple sclerosis (MS) and neuropsychiatric syndrome (NPS)) through intestinal homeostasis and neuronal function, and information exchange between the intestinal‐brain axes is regulated by miRNA–microbiota interactions in the intestinal. Created with Biorender.com. (The solid arrow in the figure represents the direct guiding effect between tissues/organs, while the dashed arrow represents the indirect guiding effect between tissues/organs.)

The rapid development of sequencing technology and the identification of an increasing number of targeted relationships between miRNAs and disease in recent years has led to increased recognition of the important role of the differential expression profiles of miRNAs and microbiota in the intestinal as biomarkers for the detection of host diseases [157] such as IBD and metabolic, immunological, functional, and behavioral disorders of the brain, and these differentially expressed miRNAs causing lesions in the host organism are also involved in the intestinal microbiota gene expression of host health regulation [12] (Figure 4). It is reported that miRNA–microbiota interactions may exert unexpected effects on disease monitoring and treatment based on sequencing technology [157].

### The role of miRNA–microbiota interactions in the regulation of chronic inflammatory bowel disease

IBD is a group of complex intestinal inflammatory diseases, including CD and UC, and the formation and remission of IBD are both closely related to intestinal homeostasis and intestinal immunity. Recent studies have demonstrated that miRNAs are closely associated with the onset and progression of IBD and that miRNAs are involved in the regulatory mechanisms of host–microbiota interactions. Specifically, miRNA–microbiota interactions can regulate host intestinal homeostasis, epithelial cell integrity, and immune response, the expression of genes related to immune cell activation and inflammatory mediator production, and the structure and function of the intestinal microbiota community to influence the development of IBD [12, 58, 158]. The intestinal microbiota is significantly altered in patients with IBD, and miRNAs can be involved in the pathogenesis of IBD through interactions with the microbiota. Interactions between miR‐29, miR‐30c, miR‐93, miR‐106b, miR‐130a, miR‐199a‐5p, miR‐515‐5p, miR‐548ab, and miR‐1226 and their potential target microbiota components that include *F. nucleatum*, *E. coli*, and segmented *filamentous bacteria* can promote the growth of these microbiota, leading to an imbalance in intestinal homeostasis and thereby exacerbate intestinal inflammation [5, 124, 159]. Similarly, the targeted binding relationship that exists between miR‐21 and a wide range of microbiota significantly reduces intestinal abundance of *Clostridium* species and *Firmicutes* abundance while increasing *Bacteroidetes* phylum abundance in patients with IBD. This is not indicative of the promotion of intestinal health but rather that miR‐21 can serve as a biomarker of intestinal homeostasis in patients with IBD [151, 160, 161]. The interactions of miR‐148‐3p and miR‐27‐3p with *Proteobacteria* have not been demonstrated to exert a direct effect on microbiota growth status but are strongly correlated with the onset of IBD that may be related to an impaired intestinal immune barrier and disrupted intestinal homeostasis [76]. Interaction between miR‐29b‐2‐5p, miR‐128, and miR‐155 and *Salmonella* facilitates bacterial invasion of cells and the release of pro‐inflammatory factors that, in turn, cause local or overall immune activation in the intestine. Additionally, miRNA–microbiota interactions affect macrophage recruitment and phagocytosis, which are detrimental to the maintenance of intestinal homeostasis and the alleviation of IBD [34, 109, 144, 162]. The regulatory effects of *Listeria monocytogenes* in the intestine on miRNA‐let‐7a1, miR‐16, miR‐29, miR‐125a‐3p/5p, miR‐145, miR‐146a, miR‐146b, miR‐149, and miR‐155 alter intestinal immunity and intestinal homeostasis, and this, in turn, can exacerbate or alleviate IBD [28, 32, 163, 164]. In contrast, miR‐155, miR‐150, miR‐143, and miR‐233 interaction with *Lactobacillus fermentum* is beneficial for remodeling intestinal barrier function, modulating intestinal inflammation, and maintaining intestinal homeostasis, all of which positively regulate intestinal homeostasis in the context of IBD. Therefore, strategies to alleviate or ameliorate IBD symptoms by artificially interfering with the interactions of miRNA microbiota within the intestinal miRNA of a patient may prove effective. Although the emergence of various types of probiotics and postbiotics has alleviated some of the intestinal disorders, their use may prove to ultimately be detrimental to intestinal homeostasis. More information regarding the regulation of miRNA–microbiota interactions in the context of IBD is presented in Table 2 [5, 76, 124, 151, 159, 160, 161, 165, 166, 167, 168].

**TABLE 2:** MiRNAs with potential target‐regulatory effects and their specific target microorganisms.

MiRNAs	Potential target microorganisms	Reference
miR‐29, miR‐30c, miR‐93, miR‐106b, miR‐130a, miR‐199a‐5p, miR‐515‐5p, miR‐548ab and miR‐1226	*F. nucleatum* *E. coli*	[5, 124, 159, 165]
	*Segmented filamentous bacteria*	
miR‐148‐3p and miR‐27‐3p	*Proteobacteria*	[76]
miR‐21	*Akkermansia muciniphila* *Clostridia*	[151, 160, 161]
	*Bacteroidetes*	
miR‐7b and miR‐7c	*Proteus*	[151]
miR‐192 and miR‐194	*Bacteroides*	[151, 166]
miR‐7g‐5p, miR‐107 and miR‐189‐3p	*Proteus*	[166]
miR‐21‐5p	*Yeast fungus*	[167]
miRNA‐let‐7b and miRNA‐let‐7c	*Parabacteroides*	[151]
miR‐155, miR‐150, miR‐143 and miR‐233	*Lactobacillus fermentum* and *Lactobacillus salivarius*	[168]
miR‐29a, miR‐128 and miR‐155	*Salmonella*	[34, 109, 144, 162]
miRNA‐let‐7a1, miR‐16, miR‐29 miR‐125a‐3p/5p, miR‐145, miR‐146a, miR‐146b, miR‐149 and miR‐155	*Listeria monocytogenes*	[28, 32, 163, 164]
miR‐194‐5p and miRNA‐let‐7c‐5p	*Enterobacteriaceae*	[76]

### The role of miRNA–microbiota interactions in the regulation of metabolic diseases

The intestine is a major site for nutrient absorption and metabolism, and the microbiota in the intestine have established a stable symbiotic relationship with the host during long‐term evolution, where the activities of microbiota exert a direct impact on the efficiency of digestion, absorption, and utilization of nutrients by the host. Dysregulation of intestinal microbiota homeostasis and the activation of the immune system caused by miRNA–microbiota interactions are important causes of metabolic disorders and disease formation in the body [169, 170, 171]. Guo et al. [172] demonstrated that miRNA‐10a‐5p regulates insulin resistance and glucose metabolism disorders induced by a high‐fat diet in mice and prevents the development of diabetes and hypertension. MiRNA‐10a‐5p also exerted a positive effect on regulating the circadian patterns of *Oscillospira*, *Ruminococcus*, and *Lachnospiraceae* in the intestine. However, the study did not assess if there was a synergistic effect between miRNAs and microbiota. Dong et al. [173] observed that the polysaccharides unique to *Polygonatum kingianum* Collett & Hemsl could regulate the structure and type of intestinal microbiota related to the metabolism of sugars and fats by downregulating the expression of miR‐122 in rats fed a high‐fat diet and prevented the development of diseases related to glycolipid metabolism in rats. Treatment with *P*. *kingianum* extract altered the expression of 29 microbiota and 27 miRNAs in the rat intestine and coregulated glycolipid metabolism through a targeting relationship. The abundance of *Parabacteroides* and *Bacillus* species was correlated with miR‐122‐5p, miR‐184, miR‐378b, and miR‐484 levels. The authors emphasized that *Parabacteroides* and miR‐484 are central members of the overall targeting relationship. A large number of microbiota and miRNAs are involved in the homeostasis of glucose–lipid metabolism and the regulation of insulin levels [174, 175], and some microbiota such as *Bacteroides* and certain miRNAs such as miR‐30d, miR‐122, and miR‐221 have been used as markers for the diagnosis of disorders of glucose–lipid metabolism [176, 177]. Our previous studies revealed targeted interactions of noncoding RNAs with nutrient transporter genes to regulate nutrient transport and absorption, but the interactions between bacterial flora and noncoding RNAs have not been addressed in previous studies [178, 179]. Therefore, we hypothesize that nutrient digestion in the intestinal tract activates the targeted binding of miRNAs to intestinal nutrient transporter genes and proteins through the intermediary action of microbiota, and this further regulates nutrient utilization efficiency and host metabolic processes that would potentially be the best means of treating metabolic diseases. Additionally, when humans and dairy animals are in a state of negative energy balance, the body will mobilize body fat to make up for the energy deficit, and excessive fat mobilization will induce nutritional metabolic disorders and cause ketosis [180]. Negative energy balance of the body will further affect the process of intestinal utilization of other nutrients and the maintenance of intestinal homeostasis, and intestinal homeostasis dysregulation is often accompanied by differential expression of a large number of miRNAs and stimulates miRNA‐microbiota interactions to regulate the intestinal barrier, intestinal immunity, and bacterial flora structure and diversity.

Compared to humans and experimental animals, ruminants possess more complex physiological structures and digestive characteristics and are affected by objective factors, and based on this, there is a lack of direct evidence to elucidate the regulation of ketosis by miRNA‐microbiota interactions. However, it can be determined that miRNA‐microbiota interactions are associated with ketosis in dairy animals and hypocalcemia and chondrodystrophies resulting from abnormalities in host calcium and phosphorus metabolism [181], and subacute acidosis of the rumen, diarrhea, hoof‐lobe inflammation, and decreased milk fat percentage are associated with abnormalities in volatile fatty acid metabolism [182].

Exploring miRNA–microbiota interactions in the intervention and treatment of metabolic diseases is an important topic for future research. However, further delineation of the composition of miRNAs and metabolites in the intestinal tract is currently not possible due to limitations in research tools, and it is important to emphasize that studies in humans must also focus on the metabolic consequences of individual differences, whereas bulk studies in animals can largely avoid these confounding factors. Moreover, personalized intervention of metabolism in omnivores is also not possible worldwide, and therefore, the construction of miRNA–microbiota–metabolite regulatory networks using multi‐batch experiments and bioinformatics and large‐scale validation are necessary, as they will contribute to targeted drug development and clinical testing.

### Regulation of brain function and behavior by miRNA–microbiota interactions

The bidirectional neurohumoral communication system between the intestines and the brain is known as the intestinal microbiota–brain axis, and studies have confirmed that this bidirectional communication may be closely related to the body's metabolism, endocrine and immune activities, neurological development, and intestinal homeostasis [183, 184, 185]. Behavioral studies suggest that the formation of this two‐way communication mechanism is related to brain functions and behaviors such as social fear, cognitive impairment, expression of fear, and the stress response [186, 187]. In recent years, increasing evidence has demonstrated that intestinal flora and miRNAs can regulate brain development and function through the intestinal–brain axis, and this, in turn, affects host brain function and behavior, as miRNA–microbiota interactions are not only manifested in the regulation of intestinal health, organismal inflammation, and nutrient metabolism but also in the modulation of the synthesis of neurotransmitters and the development of the nervous system [187]. Therefore, miRNA–microbiota interactions play important cross‐regulatory roles in body metabolism, intestinal immunity, intestinal homeostasis, and microbiota–intestinal–brain axis‐related diseases, including alzheimer's disease (AD), parkinson's disease (PD), major depressive disorder (MDD), schizophrenia (SCZ), bipolar disorder (BD), autism spectrum disorder (ASD), attention deficit hyperactivity disorder (ADHD), multiple sclerosis (MS), and neuropsychiatric disorders (NPD) [188, 189, 190, 191, 192, 193]. It can be observed that miRNA‐microbiota interactions play a dominant role in the information exchange of the intestinal‐brain axis (Figure 4).

MiR‐132 plays a key role in regulating brain synaptic plasticity and memory formation, as it binds to targeted inositol‐triphosphate 3‐kinase B (InsP3KB) and enhances human cognitive ability by bidirectionally regulating the deposition of internal and extracellular proteins [194]. Targeted binding of miR‐132 to nuclear receptor subfamily 4 group A member 2 (NR4A2) is an important pathway regulating the development of midbrain neurons and brain behavior [195]. Additionally, the effects of miR‐132 on *FOXO3a* and phosphatase and tensin homolog also directly modulate and accelerate the apoptosis of neuronal cells. In mice, providing miR‐132 mimics AD in the brain and inhibits neural function in the hippocampus by rapidly decreasing the brain Aβ40‐42 levels and tau hyperphosphorylation and inhibiting neural function in the hippocampus [194, 196, 197]. Kim et al. [142] noted that targeted binding of miR‐132 to *FOXO3a* in the intestine is involved in intestinal immunity and autophagy processes in IEC. This suggests that the expression of miRNA‐132 may be involved in intestinal microbiota–brain axis regulatory activities through bidirectional neurohumoral communication. Similarly, in the nervous system, miR‐7 can further induce neuroinflammation in Parkinson's disease through protein‐mediated expression of organismal inflammatory factors [198]. Injection of miR‐7 into the basal ganglia of the mouse brain alleviates this process and reduces NLRP3 activation and preserves a portion of dopaminergic neurons [199]. In the intestine, the main role of miR‐7 is to regulate the intestinal barrier and intestinal immunity by altering the roles of *NF‐kB*, *ERK*, and *AKT* signaling [19]. Additionally, intestinal microbiota‐derived toxins can further stimulate the immune system by targeting miRNA‐146a and miRNA‐155, thereby activating the body's immunity and transmitting disease‐causing signals to the brain through the microbiota–brain axis. After receiving the signal, the brain can specifically guide downregulated miRNA‐146a and miRNA‐155 to target binding with mRNA and assist innate immunity to participate in the immune response, whereas the remaining upregulated miRNAs bind with the corresponding mRNA (complement factor H) and directly trigger neurological diseases, thus affecting brain function and behavior [200].

In summary, miRNA–microbiota interactions contribute to the organic connection between the intestinal and brain neurons, forming an intestinal microbiota–brain situation in which both the intestine and the brain can either benefit or be adversely affected. Disruption of intestinal function leads to abnormal brain function and behavior, and brain dysfunction can disrupt the homeostatic pattern of the intestine. A vicious circle can develop between the intestine and the brain, which can have adverse effects on physical and mental health. A healthy intestinal environment creates a healthy brain, and a healthy brain creates a healthy intestinal environment. Therefore, miRNA–microbiota interactions are an important tool for regulating brain function and behavior.

## 5 PROSPECTIVE ANALYSIS OF miRNA–MICROBIOTA INTERACTIONS FOR PERSONALIZED REGULATION OF INTESTINAL HOMEOSTASIS

**FIGURE 5:**
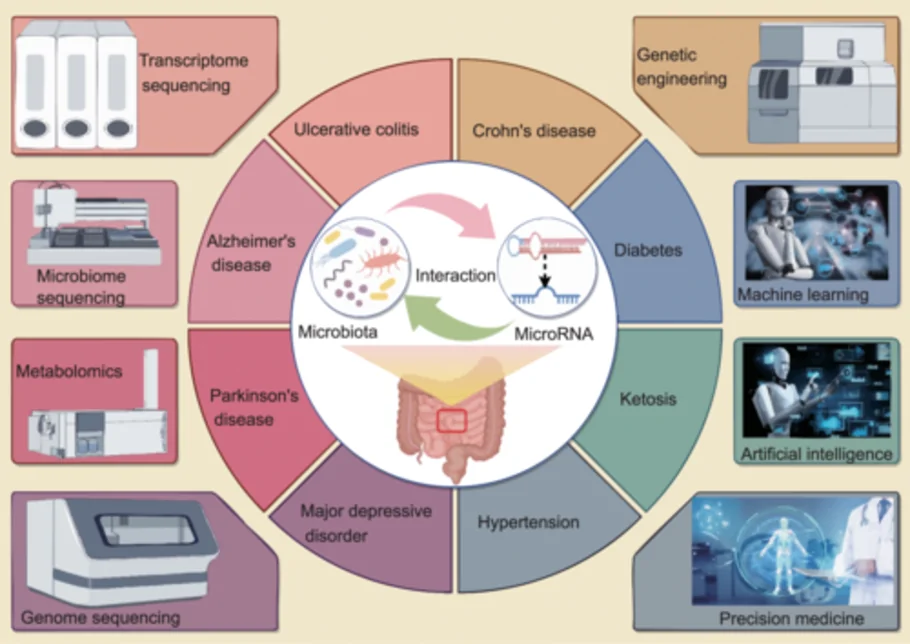
Prospective analysis of miRNA–microbiota interactions research. MiRNA–microbiota interactions further intervene in host health through the regulation of intestinal homeostasis, but the exact direction and extent of their regulation is unknown. Future studies should fully integrate multi‐omics and artificial intelligence technologies to further elucidate the clinical applications of positively utilizing miRNA–microbiota interactions for host health. Created with Figdraw.com.

With the support of sequencing technology to sequence and analyze miRNAs and microbiota from different groups and different health states, the correlations among them are beginning to be revealed, and their regulatory mechanisms in the context of intestinal homeostasis can be further investigated. Meanwhile, based on the approach of multi‐omics joint analysis, the integrated study of miRNA–microbiota interactions with host metabolism and immunity is anticipated to reveal individual differences and the mechanisms underlying related diseases [201]. Current integrative analyses across multiple omics disciplines have highlighted the significant potential for elucidating the interactions between miRNAs and the microbiota. Specifically, to thoroughly investigate these interactions, we can employ genomic sequencing to swiftly identify the genetic backgrounds of miRNAs, transcriptomic sequencing to analyze alterations in miRNA expression, and proteomic sequencing to assess the impacts of miRNAs on protein expression. Additionally, metagenomic sequencing can be utilized to explore the structure and functionality of microbiota communities, while metabolomic sequencing helps reveal how miRNAs modulate the composition and functionality of the microbiota via metabolic pathways. Moreover, advanced techniques such as single‐cell genomics, single‐cell transcriptomics, single‐cell proteomics, and single‐cell metabolomics can be applied to explore the interactions between miRNAs and microbiota at the single‐cell level. Following this, extensive sequencing data can be analyzed quickly through cutting‐edge artificial intelligence, bioengineering, and machine learning methodologies, thus allowing for the precise identification of therapeutic targets and the rapid elucidation and mapping of the regulatory networks involved in miRNA and microbiota interactions [202]. Ultimately, these efforts will contribute to the formulation of personalized treatment strategies for intestinal‐related disorders. Additionally, regulatory tools and therapeutics for miRNAs and microbiota will continue to be innovated and improved. In the future, researchers may develop more precise miRNA regulation technologies, such as gene editing and RNA interference for regulating the miRNA expression level of individuals [203]. Meanwhile, the regulation tools for the microbiota will be more diversified, including probiotics and prebiotics, and others, to allow for precise intervention against the microbiota [204]. Most importantly, the health benefits brought by personalized regulation of intestinal homeostasis will drive the transformation of the medical model, and we may see more personalized therapeutic solutions based on miRNA–microbiota interactions that not only provide precise treatment for intestinal‐related diseases but also enable personalized health management to help individuals achieve a healthier and more balanced intestinal microecological state [205]. Figure 5 illustrates our prospective analysis of miRNA–microbiota interaction studies.

## 6 CONCLUSION

This study summarizes the regulatory mechanisms and modes of action by which miRNAs interact with the microbiota to regulate intestinal homeostasis and host health. Although numerous studies have confirmed the important roles of individual miRNAs and microbiota in intestinal health and disease detection and treatment, further research is needed to specifically regulate the combined effects of both through the regulation of intestinal homeostasis and to develop further interventions to promote host health. None of the mechanisms mentioned in this review regarding the miRNA–microbiota interactions that regulate intestinal homeostasis and maintain the health of the body function alone, and a complex regulatory network exists among them. Therefore, when researchers face the scientific question of miRNA–microbiota interactions in regard to regulating intestinal homeostasis and body health, they should also analyze them in combination with factors such as genetic background, dietary structure, work habits, work environment, and medication history. It is evident that the use of miRNA–microbiota interactions for personalized regulation of intestinal homeostasis possesses great prospects and potential. This research direction will not only deepen the understanding of intestinal microbiota ecosystems and miRNA regulatory networks but also promise new hope for personalized medicine and the treatment of intestinal‐related diseases.

## AUTHOR CONTRIBUTIONS

**Tianle He**: Conceptualization; investigation; writing—original draft; writing—review and editing; visualization; validation; methodology; software; formal analysis; project administration; data curation. **Jiaying Ma**: Investigation; conceptualization; methodology; visualization; software; data curation; writing—review and editing. **Shuai Liu**: Visualization; writing—review and editing; project administration; formal analysis; software; methodology; investigation. **Boyan Ma**: Conceptualization; investigation; methodology; validation; formal analysis; software. **Jingtao You**: Writing—review and editing; visualization; validation; formal analysis; software; **Jingjun Wang**: validation; formal analysis; visualization; writing—review and editing. **Mengmeng Li**: Writing—review and editing; software; validation; methodology; visualization. **Wei Wang**: Methodology; validation; visualization; writing—review and editing; formal analysis. **Ya Jing Wang**: Validation; visualization; writing—review and editing; project administration; formal analysis. **Shengli Li**: Visualization; validation; writing—review and editing. **Zhijun Cao**: Conceptualization; investigation; funding acquisition; writing—review and editing; visualization; validation; methodology; formal analysis; project administration; resources; supervision; data curation.

## CONFLICT OF INTEREST STATEMENT

The authors declare no conflicts of interest.

## 1 ETHICS STATEMENT

No animals or humans were involved in this study.

## Data Availability

Data sharing is not applicable to this article as no datasets were generated or analyzed during the current study. No new data and scripts were generated for this review. Supplementary information (graphical abstract, slides, videos, Chinese translated version, and update materials) may be found in the online DOI or iMeta Science http://www.imeta.science/imetaomics/.
